# Effects of Hardwood Biochar on Methane Production, Fermentation Characteristics, and the Rumen Microbiota Using Rumen Simulation

**DOI:** 10.3389/fmicb.2019.01534

**Published:** 2019-07-10

**Authors:** Rebecca Teoh, Eleonora Caro, Devin B. Holman, Stephen Joseph, Sarah J. Meale, Alex V. Chaves

**Affiliations:** ^1^Sydney School of Veterinary Science, Faculty of Science, The University of Sydney, Camperdown, NSW, Australia; ^2^School of Life and Environmental Sciences, Faculty of Science, The University of Sydney, Camperdown, NSW, Australia; ^3^Department of Agricultural, Forestry and Food Science, University of Turin, Turin, Italy; ^4^Lacombe Research and Development Centre, Agriculture and Agri-Food Canada, Lacombe, AB, Canada; ^5^School of Materials Science and Engineering, University of New South Wales, Sydney, NSW, Australia; ^6^School of Agriculture and Food Sciences, Faculty of Science, The University of Queensland, Gatton, QLD, Australia

**Keywords:** 16S rRNA gene abundance, fungal ITS1 region, pyrolyzed biomass, ruminant feeds, RUSITEC system

## Abstract

Biochar is a novel carbonized feed additive sourced from pyrolyzed biomass. This compound is known to adsorb gasses and carbon, participate in biological redox reactions and provide habitat biofilms for desirable microbiota proliferation. Therefore, biochar holds potential to modify rumen fermentation characteristics and reduce enteric CH_4_ emissions. The objective of this study was to investigate the effect of hardwood biochar supplementation on fermentation parameters, methane (CH_4_) production and the ruminal archaeal, bacterial, and fungal microbiota using the *in vitro* RUSITEC (rumen simulation technique) system. Treatments consisted of a control diet (oaten pasture: maize silage: concentrate, 35:35:30 w/w) and hardwood biochar included at 400 or 800 mg per day (3.6 and 7.2% of substrate DM, respectively), over a 15-day period. Biochar supplementation had no effect (*P* ≥ 0.37) on pH, effluent (mL/d), total gas (mL/d), dry matter (DM) digestibility or CH_4_ production (mg/d). The addition of 800 mg biochar per day had the tendency (*P* = 0.10) to lower the % of CH_4_ released in fermentation compared to 400 mg/d biochar treatment. However, no effect (*P* ≥ 0.44) was seen on total VFA, acetate, propionate, butyric, branched-chain VFA, valerate and caproate production and the ratio of acetate to propionate. No effect (*P* > 0.05) was observed on bacterial, archaeal or fungal community structure. However, biochar supplementation at 800 mg/d decreased the abundance of one Methanomethylophilaceae OTU (19.8-fold, *P* = 0.046) and one *Lactobacillus* spp. OTU (31.7-fold, *P* < 0.01), in comparison to control treatments. Two fungal OTUs classified as *Vishniacozyma victoriae* (5.4 × 10^7^ increase) and *Sporobolomyces ruberrimus* (5.4 × 10^7^-fold increase) were more abundant in the 800 mg/d biochar samples. In conclusion, hardwood biochar had no effects on ruminal fermentation characteristics and may potentially lower the concentration of enteric CH_4_ when included at higher dosages by manipulating ruminal microbiota abundances.

## Introduction

Methane (CH_4_) mitigation strategies are of environmental, economic and food security importance. Worldwide demand for meat and dairy is expected to double by 2050 (Food and Agriculture Organization of the United Nations, 2008) with CH_4_ production predicted to exponentially increase simultaneously (Gerber, 2013). In ruminants, methane (CH_4_) production is an end-product derived from the fermentation of low-quality forages by ruminal methanogens (Deppenmeier, 2002; Broucek, 2014). This enteric fermentation accounts for 40% of global livestock sector greenhouse gas (GHG) emissions, with cattle being the main contributor of enteric CH_4_ (77%), followed by buffalos (13%) and small ruminants (10%) (Gerber, 2013). Ruminal methanogenesis represents an estimated 2–12% gross energy intake wastage (Van Haarlem et al., 2008; Ramin and Huhtanen, 2013; Broucek, 2014) and a source of livestock health, monetary and productivity loss (Chagunda et al., 2009).

The modification of dietary composition and additives are viable strategies for altering ruminal fermentation and reducing CH_4_ production (Beauchemin et al., 2008; Hristov et al., 2013; Broucek, 2014; Duarte et al., 2017; Haque, 2018). Fermentation characteristics are directly affected by the ruminal microbiota, where varied concentrations of volatile fatty acids (VFA), gas production, dry matter (DM) digestibility, and CH_4_ production are dependent on the microbial population. This has been demonstrated in both *in vitro* (Hansen et al., 2012; Leng, 2014; Henderson et al., 2015; Duarte et al., 2017; Ramos et al., 2018; Terry et al., 2018) and *in vivo* studies (Leng et al., 2012c).

Biochar is a novel carbonized feed additive sourced from pyrolyzed biomass. This compound is highly porous with a large internal surface area (Thies and Rillig, 2009) which allows it to adsorb gasses and carbon, detoxify via binding, and provide habitat biofilms for desirable microbiota proliferation (Hansen et al., 2012; Joseph et al., 2015; Lehmann and Joseph, 2015; Jeffery et al., 2016; Leng, 2017). Biochar also holds electron-mediating properties in biological redox reactions (Yu et al., 2015), and provides a benefit in terms of higher feed-conversion efficiency in ruminants and reduced GHG emissions (Leng et al., 2012a,c; Kammann et al., 2017). Therefore, it is no surprise that biochar has become a promising additive in crop soil rejuvenation and methane mitigation in livestock production which are the agricultural sectors most severely affected by drought and climate variation (Joseph et al., 2015; Lehmann and Joseph, 2015; Jeffery et al., 2016; Agegnehu et al., 2017; Food and Agriculture Organization of the United Nations et al., 2018).

We hypothesized that the addition of hardwood biochar would modify fermentation characteristics and result in a drop in CH_4_ production in the rumen. As such, the objective in this study was to investigate the effect of hardwood biochar supplementation on fermentation parameters, CH_4_ production, and the rumen microbiota using the *in vitro* RUSITEC (rumen simulation technique) system.

## Materials and Methods

This study was carried out in accordance with The University of Sydney Animal Ethics Committee (Approved Protocol number 2015/835) and housed at The University of Sydney, Corstorphine (Camden Farm Dairy, Cobbitty, NSW, Australia).

### Experimental Design and Treatments

*In vitro* ruminal fermentation was conducted using a RUSITEC unit to evaluate the effect of hardwood biochar supplementation on methane production, fermentation characteristics and the rumen archaeal, bacterial, and fungal microbiota. The study was conducted over a 30-day period composed of two runs of 15 days. Each run was made up of 6 days of adaptation followed by 9 days of sampling. The experiment was a completely randomized design with three treatments and two replicates per treatment. The three treatments consisted of a control diet (CON, no biochar addition), a diet supplemented with 400 mg biochar/d (added at 3.6% of substrate dry matter (DM) per day), and another diet with 800 mg biochar/d (7.2% of substrate DM per day).

Fermentation parameters (total gas, CH_4_, pH and effluent volume production) underwent daily collection over the experimental period. Effluent samples were collected from day 7 to the end of each 15-day run. Nylon bags were retained for DM digestibility on days 8, 9, and 11–14, and liquid-associated microbes (LAM) and solid-associated microbes (SAM) were sampled on days 0, 5, 10, and 15.

### Preparation of Substrate, Biochar and Rumen Inoculum

The basal substrate diet consisted of an oaten pasture:maize silage:concentrate (35:35:30 w/w) ratio that was weighed into nylon bags (11 cm × 6.5 cm, pore size 150 μm) for a total mass of 11 g of substrate (DM basis). All three substrate components were derived from the Corstorphine farm (Cobbitty, NSW, Australia). Plant material of oaten (*Avena sativa*) was collected on 12th January, 2017 at the Corstorphine Farm research site of the University of Sydney, Camden Campus, NSW, Australia (34° 04′ S; 150°81 69′E). The climate was warm-temperate with a mean annual minimum and maximum temperature of 10.7 and 23.3°C, respectively. The annual average rainfall was 738 mm (1900–2010). Multiple oaten samples were randomly selected and harvested at grazing height ≥5 cm above ground level to mimic grazing by cattle. Maize silage was randomly sampled from different locations from across the silo pit face. Feed components were processed immediately upon return to the laboratory (within 45 min of collection). This involved oven drying at 55°C, grinding and then passage through a 4 mm sieve using a feed mill (Model: Cutting Mill SM100, Retsch, Haan, Germany). Dried samples were then kept at room temperature until the day of incubation.

The final substrate consisted of 90.47% DM, 10.5% crude protein, 35.2% non-fibrous carbohydrates, 2% ether extract, 45.8% neutral detergent fiber and 6.5% ash (DM-basis). The soluble DM across all treatments was 26.2 ± 0.91 (mean ± SD). The basal diet was analyzed according to the Association of Official Analytical Chemists [AOAC] (2006) methods for DM (method 967.03), ash (method 942.05), ether extract (EE; method 920.29), and crude protein (CP; method 990.03). Neutral detergent fibre (NDF) content was determined by methods outlined by Van Soest et al. (1991) using sodium sulfite and heat stable a-amylase. Lastly, non-fibrous carbohydrate (NFC) concentration was determined as the percentage of organic material remaining according to the equation: NFC (% DM) = 100 – (CP + NDF + EE + ash) (Mertens, 1997).

The biochar product used was a novel mineral-activated biocarbon created by Dr. Stephen Joseph based on his experience working with cattle farmers (Joseph et al., 2015). The base material was sourced from hardwood black butt sawdust, with additives of bentonite, zeolite, urea, ferrous sulfate (FeSO_4_.7H_2_O), rock phosphate, straw and basalt dust obtained from Borals Dunmore quarry (Dunmore, NSW, Australia). The organic material underwent slow pyrolysis at 650°C with a hold time of 1 h. Biochar was characterized via soil analysis performed by New South Wales Department of Primary Industries (DPI, NSW, Australia), and the results are presented in Table 1. The biochar soluble DM fraction was 35.3%; that is, 35.3% of the total amount of biochar added in each treatment was left in the bags once in solution. Since biochar is not digested by rumen microbes, the remaining 64.7% (insoluble DM fraction) of the amount added by the treatments were discounted for DM digestibility calculations.

**Table 1:** Chemical composition and physical properties of hardwood biochar used.

Component	Property	Component	Property
Electrical conductivity, dS/m	1.3	Water soluble phosphorus, mg/kg	6.3
pH	8.2	Aluminum, mg/kg	15,000
Total carbon, %	10	Arsenic, mg/kg	<5
Total nitrogen, %	0.2	Boron, mg/kg	19
Acid neutralizing capacity, % CaCO_3_ equivalent	6.5	Calcium, %	2.7
Total organic carbon, %	10	Cadmium, mg/kg	0.42
Bray phosphorus, mg/kg	4.5	Cobalt, mg/kg	6.5
KCl extraction ammonium-n, mg/kg	3.4	Chromium, mg/kg	21
KCl extraction nitrate-n, mg/kg	0.38	Copper, mg/kg	25
Aluminum, cmol(+)/kgˆ	<0.1	Iron, mg/kg	24,864
Calcium, cmol(+)/kgˆ	13	Potassium, %	0.42
Potassium, cmol(+)/kgˆ	2.6	Magnesium, %	0.4
Magnesium, cmol(+)/kgˆ	0.75	Manganese, mg/kg	450
Sodium, cmol(+)/kgˆ	7	Molybdenum, mg/kg	<1
CEC (effective), cmol(+)/kgˆ	23	Sodium, %	0.63
Calcium:Magnesium	17	Nickel, mg/kg	7
Exchangeable calcium, % of ECEC^∗^	55	Phosphorus, %	0.88
Exchangeable potassium, % of ECEC^∗^	11	Lead, mg/kg	7.8
Exchangeable magnesium, % of ECEC^∗^	3.3	Sulfur, %	0.89
Exchangeable sodium percentage, % of ECEC^∗^	30	Selenium, mg/kg	<4
Formic acid soluble phosphorus, mg/kg	6,900	Zinc, mg/kg	66


*ˆcmol(+)/kg, centimoles of positive charge per kg of soil. ^∗^ECEC, effective cation exchange capacity.*

On the first day of each experimental run, rumen inoculum was collected from one ruminally fistulated non-lactating Holstein dairy cow. The donor cow was fed pasture *ad libitum*, including oats supplemented with maize silage (5 kg DM/d) and oaten hay (2 kg DM/d).

The inoculum was collected 3 h post-morning feeding, and solid and liquid portions of the rumen digesta were separated using cheesecloth filtration. Each portion was saved separately within preheated thermos containers, and immediately transported to the laboratory for the initial RUSITEC inoculum.

### RUSITEC Fermentation Procedure

One RUSITEC apparatus was set up with six fermentation vessels (800 mL capacity each) submerged in a 39°C water bath. At the start of each experiment run, each fermentation vessel was filled with 780 mL of ruminal fluid and contained a smaller inner vessel which held two nylon bags (11.5 cm × 7.5 cm, pore size 150 μm). One bag contained approximately 70 g of wet weight of rumen solids, and the other bag held one of the three experimental treatments according to the randomized design (i.e., control, 400 mg biochar or 800 mg biochar).

Each fermenter vessel had an input port for buffer infusion and an outlet port for effluent. These vessels were constantly infused with McDougall’s buffer at a dilution rate of 33 mL per h. An electric motor was used to continuously move the inner vessels up and down to mimic rumination and mixing of particles and ruminal fluid. After 24 h of incubation, the rumen solids bag in each vessel was replaced with a new nylon bag containing the corresponding experimental treatment. This new bag replacement would continue every 24 h onward until day 15, with the removal of the nylon bag that has been incubating for 48 h. Therefore two bags would be present in each vessel at any given. Bags collected at day 15 were not used in DM digestibility calculations as only 24 h of incubation occurred.

### Sample Collection and Analysis

#### Gas Production and Methane Production

Daily gas production was collected into air-tight bags (Plastigas, Linde AG, Munchen, Germany) connected to the effluent flasks. The total volume of gas produced by each fermenter vessel was then determined by connecting the gas bag to a drum-type gas meter (Terry et al., 2018) and evacuating gas by applying manual pressure to the bag. Daily gas production was expressed as mL/day.

Methane (CH_4_) production was determined from day 7 until the end of each 15-day run. Prior to measurement of daily gas production and evacuation of gas bags, 12 ml of gas was removed from each gas bag, before sampling 17 mL from each gas bag using a 20 mL syringe. This 17 mL gas sample was then transferred into a 10 mL evacuated exetainer (Labco Ltd., High Wycombe, United Kingdom). A 3 mL subsample was taken from the exetainer and CH_4_ concentration was determined by gas chromatography using a gas chromatograph Agilent model 7890A. The settings used were the flame ionization detector (FID) set up at 60°C, air flow 300 mL per min and makeup flow (N_2_) running at 30 mL per min installed with a capillary column (Restek Rt-Q-Bond, 30 m × 0.53 mm ID × 20 μm). Helium (H_2_) was used as a carrier gas at 30 mL per min. The Splitless inlet was heated to 60°C, and set at 9.526 PSI, H_2_ total flow 33 mL per min, septum purge flow 3 mL per min. The oven temperature was set to 60°C. CH_4_ production was calculated by multiplying the total gas volume by the percentage of CH_4_, with correction for temperature and pressure (Duarte et al., 2017). Results were expressed as mg CH_4_/g DM.

#### Dry Matter (DM) Digestibility and pH Determination

*In vitro* DM digestibility was determined using the digested substrate remaining within each nylon bag after 48 h of fermentation. Collection of these bags took place on days 8 and 10–13, when DNA extraction from bags was not required. After removal from each vessel, each nylon bag was gently squeezed, and residual buffer was replaced into the vessel to retain solid-phase-associated microorganisms. Each bag was then washed using a washing machine set at cold delicate clothes cycle for 30 min, before being dried at 60°C until a constant weight was obtained (approx. 48 h). The residue weight was recorded and used in the calculation of DM digestibility (DMD) which was expressed as a percentage (%) of the original DM substrate excluding the biochar.

DMD (%) = [digestible DM (g)/total substrate incubated excluding biochar (g)] × 100

Digestible DM (g) = total substrate incubated excluding biochar (g) – [residue weight (g) after 48 h of incubation – biochar insoluble DM fraction (g)]

Biochar insoluble DM faction (g) = 0.647 × amount of biochar in each treatment (0, 0.4 or 0.8 g)

Fermenter vessel fluid pH was measured daily during bag exchange using a pH meter (TPS pH-mV-Temp Meter, Model WP-80) calibrated at 39°C.

#### Effluent Volume and Volatile Fatty Acids (VFA) Production

Effluent production was determined by collection into 2 L glass flasks submerged in ice to halt fermentation and microbial growth. The effluent volume production was measured daily using a measuring cylinder and expressed as mL/day.

From days 7–15, effluent contents were transferred into 2 mL centrifuge microtubes. The supernatant was centrifuged at 13,500 × *g* for 2 min at 5°C for the determination of volatile fatty acids. A 1.5 mL subsample of the supernatant was transferred into 2 mL centrifuge tubes and acidified with 0.3 mL of metaphosphoric acid (0.20; wt/v). The subsample was then frozen at -20°C until it was analyzed for VFA concentrations using a gas chromatography (GC; Agilent model 7820A). The settings used were FID set up at 250°C, air flow 300 mL per min, makeup flow (N_2_) ran at 30 mL per min with a capillary column (DB-FFAP, 30 m × 0.32 mm ID × 1 μm). Helium was used as a carrier gas with a flow rate of 30 mL per min. Split inlet was heated to 225°C, and set at 9.526 PSI, with H_2_ constant flow 1.5 mL per min, and split ration 50:1. The oven temperature was programmed to 150°C (hold 1 min) and 5°C per min to 195°C (hold 3 min). Daily total and individual VFA production were estimated by multiplying VFA concentration by the volume of effluent. In RUSITEC run 1, VFA samples of day 7–8 were lost.

#### Sequencing and Analysis of the Archaeal and Bacterial 16S rRNA Gene and the Fungal ITS1 Region

The V4 region of the archaeal and bacterial 16S rRNA gene was amplified as previously described (Terry et al., 2018). The primers ITS1F (5′-CTTGGTCATTTAGAGGAAGTAA-3′) and ITS2 (5′-GCTGCGTTCTTCATCGATGC-3′) (Buee et al., 2009) were used to amplify the ITS1 region of fungi. Both 16S rRNA gene and ITS1 sequences were sequenced using the MiSeq Reagent Kit v2 (500 cycles; Illumina, Inc., San Diego, CA, United States) and an Illumina MiSeq instrument according to manufacturer’s instructions.

The 16S rRNA gene and ITS1 region sequences were processed using DADA2 v. 1.8 (Callahan et al., 2016) in R v. 3.5.1. Briefly, the forward and reverse 16S rRNA gene sequences were trimmed to 220 and 200 bp, merged, and then chimeras were removed. Taxonomy was assigned to the remaining sequences, referred to here as operational taxonomic units (OTUs) at 100% similarity, using the RDP naïve Bayesian classifier and the SILVA SSU database release 132 (Quast et al., 2012). For the fungal ITS1 sequences, the reads were quality-filtered using the default parameters in DADA2 with a minimum sequence length of 50 bp required, however, the reads were not trimmed to the same length due to the variability in the length of the ITS1 reads. The quality-filtered reads were then merged, chimeras removed, and taxonomy assigned to the ITS1 sequences using the RDP naïve Bayesian classifier and the UNITE database v. 8.0 (Koljalg et al., 2013). Overall, 84.8% of the ITS1 sequences were assigned to a fungal species. The number of OTUs per sample, Shannon diversity index, and inverse Simpson’s diversity index for 16S rRNA gene and ITS1 datasets were calculated in R using Phyloseq v. 1.26.0 (McMurdie and Holmes, 2013). Bray-Curtis dissimilarities were calculated using vegan 2.5–3 (Oksanen et al., 2013) in R and the effect of Biochar concentration was assessed using PERMANOVA (adonis function). Prior to analysis of the diversity metrics and Bray-Curtis dissimilarities, the 16S rRNA gene and ITS1 samples were randomly subsampled to 27,500 and 9,100 sequences per sample, respectively, which corresponded to the lowest number of sequences per sample. DESeq2 v. 1.18.0 (Love et al., 2014) was used to identify differentially abundant OTUs between the 0 and 800 mg/L samples (false discovery rate <0.05). Only those OTUs present in at least 25% of the samples analyzed were included and the samples were not randomly subsampled for this analysis. All 16S rRNA gene and ITS sequences were submitted to the Sequence Read Archive under BioProject number PRJNA525436.

### Statistical Analysis

Ruminal fermentation data was analyzed using the MIXED procedure of SAS (SAS Inc., 2019; SAS Online Doc 9.1.3. Cary, NC, United States). The total number of samples analyzed for each treatment was 36 [e.g., 9 days sampling × 4 fermenters per treatment (2 fermenters per run × 2 runs)]. Means were compared using the least square mean linear hypothesis. The model included the fixed effects of treatment, day and treatment by day interactions with the day of sampling from each fermenter treated as a repeated measure. Therefore, the individual fermenter was used as the experimental unit for statistical analysis. The minimum values of AIC (Akaike’s Information Criterion) were used to select the covariance structure among compound symmetry, heterogeneous compound symmetry, autoregressive, heterogeneous autoregressive, Toeplitz, unstructured and banded for each parameter. Significance was declared at *P* ≤ 0.05 and a trend was discussed when 0.05 < *P* ≤ 0.10. Only when effect of treatment was significant (*P* ≤ 0.05), orthogonal polynomial contrasts were performed to test for linear and quadratic responses to increasing concentration of biochar (0, 400, and 800 mg/d) in the substrate.

## Results

### Effect of Biochar on Dry Matter Digestibility, VFA, pH, Effluent, Total Gas and CH_4_ Production

The addition of biochar had no effect (*P* ≥ 0.37) on pH, effluent, total gas or CH_4_ production (mg/d) (Table 2). A tendency was identified (*P* = 0.10) where the supplementation of 800 mg biochar/d lowered the % of CH_4_ in the gas sample (expressed as a percentage of total gas) compared to 400 mg biochar/d treatment. Biochar supplementation had no (*P* ≥ 0.44) effect on DM digestibility (Table 2), total and individual VFA production (Table 3).

**Table 2:** Effect of biochar on pH, effluent, total gas, CH_4_ concentration and amount and dry matter (DM) digestibility produced over a 24 h period in a RUSITEC system, with a mixed hay-silage-concentrate diet.

	Biochar (mg/d)				*P*-value				
			
	Control, 0	400	800	SEM	Treat	Day	Treat × Day	L	Q
pH	6.75	6.76	6.76	0.010	0.53	0.01	0.88	0.37	0.51
Effluent, mL/d	739	744	742	7.1	0.87	<0.01	0.99	0.76	0.68
Total gas, mL/d	1230	1365	1302	67.7	0.37	0.07	0.98	0.46	0.24
CH_4_, %	3.90ab	3.97a	3.60b	0.117	0.10	0.58	0.96	0.09	0.15
CH_4_, mg/d	35.8	39.6	35.5	1.73	0.20	<0.01	0.88	0.91	0.08
Dry matter digestibility^1^, %	66.2	66.4	65.8	0.65	0.82	0.87	0.73	0.67	0.64


*SEM, standard error of the means; L, linear; Q, quadratic.^*1*^DMD (%) = [digestible DM (g)/total substrate incubated excluding biochar (g)] × 100. Digestible DM (g) = total substrate incubated excluding biochar (g) – [residue weight (g) after 48 h of incubation – biochar insoluble DM fraction (g)]. Biochar insoluble DM faction (g) = 0.647 × amount of biochar in each treatment (0, 0.4 or 0.8 g).*

**Table 3:** Effect of biochar on individual volatile fatty acids (VFA) production over a 24 h period in a RUSITEC system, with a mixed hay-silage-concentrate diet.

	Biochar (mg/d)				*P*-value				
			
	Control, 0	400	800	SEM	Treat	Day	Treat × Day	L	Q
**Volatile fatty acids, mmol/d**								
Total VFA	29.9	30.4	29.9	1.37	0.96	0.04	0.30	0.98	0.77
Acetate (A)	11.8	12.1	12.1	0.73	0.96	0.43	0.31	0.81	0.90
Propionate (P)	8.2	8.2	7.9	0.29	0.66	0.02	0.39	0.48	0.58
Butyric	4.6	4.6	4.6	0.35	0.98	<0.01	0.30	0.89	0.92
BCVFA	2.06	2.03	1.99	1.82	0.86	0.01	0.07	0.59	0.98
Valerate	2.92	3.05	2.95	0.071	0.44	<0.01	0.25	0.77	0.23
Caproate	0.36	0.39	0.43	0.050	0.61	<0.01	0.95	0.33	0.99
Ratio A:P	1.45	1.47	1.54	0.082	0.77	0.06	0.14	0.50	0.83


*SEM, standard error of the means; L, linear; Q, quadratic; BCVFA, branched-chain volatile fatty acids (iso-butyrate+iso-valerate).*

### Effect of Biochar on the Rumen Archaeal and Bacterial Microbiota

**FIGURE 1:**
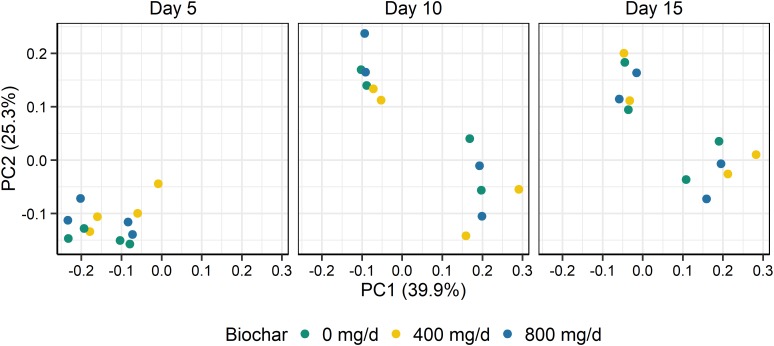
Principal coordinates plot (PCoA) of the Bray-Curtis dissimilarities for the archaeal and bacterial solid-associated microbe (SAM) samples by sampling time and biochar concentration. The percentage of variation explained by each principal coordinate are indicated on the axes.

**FIGURE 2:**
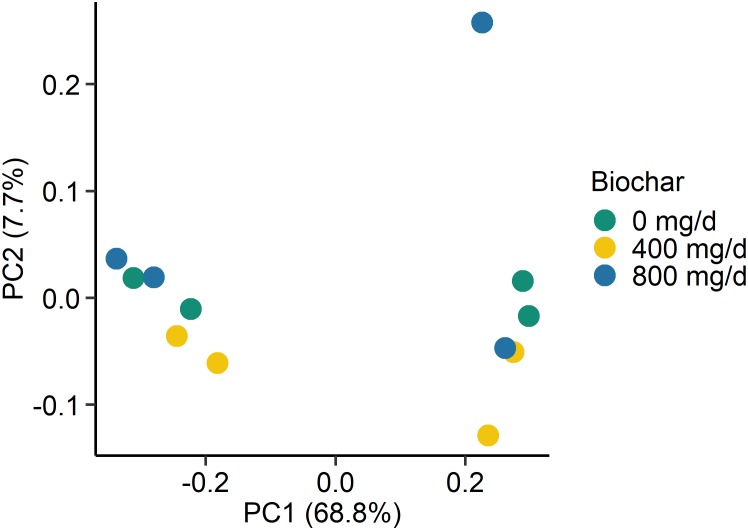
Principal coordinates plot (PCoA) of the Bray-Curtis dissimilarities for the archaeal and bacterial liquid-associated microbe (LAM) samples by biochar concentration. The percentage of variation explained by each principal coordinate are indicated on the axes.

Supplementation with biochar had no significant effect on the rumen microbial community structure for both the SAM (*P* > 0.05; Figure 1) and LAM samples (*P* > 0.05; Figure 2) at all three sampling times, indicating that the most abundant taxa were unaffected by the biochar. Likewise, there were no OTUs that were differentially abundant between the 0 mg/d and 800 mg/d biochar treatments at days 15 in the LAM samples (*P* > 0.05). Within the SAM samples, the abundance of one OTU classified at the family level as Methanomethylophilaceae was 19.8-fold higher in the 0 mg/g vs. 800 mg/d biochar treatment (*P* = 0.046), as was a *Lactobacillus* OTU that was 31.7-fold greater in the 0 mg/g vs. 800 mg/d biochar samples at d 15 (*P* < 0.01). Both the SAM and LAM samples were dominated by *Megasphaera* and *Prevotella* spp. (Supplementary Figures S1, S2). The SAM samples also had a high relative abundance (>5%) of *Bifidobacterium, Fibrobacter*, and *Lactobacillus* spp. The microbial richness and diversity of both the LAM and SAM samples was not affected by the addition of biochar (Table 4; *P* > 0.05).

**Table 4:** Effect of biochar on archaeal and bacterial richness and diversity in a RUSITEC system, with a mixed hay-silage-concentrate diet.

	Biochar (mg/d)				*P*-value		
			
	Control, 0	400	800	SEM	Treat	L	Q
**Liquid-associated microbes**							
Day 15							
Number of OTUs	436.8	481.5	490.3	37.55	0.58	0.34	0.70
Shannon diversity	4.47	4.48	4.64	0.310	0.91	0.71	0.85
Inverse Simpson’s diversity	37.3	33.1	46.6	16.34	0.84	0.70	0.67
**Solid-associated microbes**							
Day 5							
Number of OTUs	225.3	253.3	268.5	22.62	0.43	0.21	0.82
Shannon diversity	2.81	3.06	3.02	0.132	0.39	0.28	0.39
Inverse Simpson’s diversity	5.1	6.4	6.0	0.39	0.11	0.13	0.12
Day 10							
Number of OTUs	253.5	233.8	251.8	17.85	0.70	0.94	0.41
Shannon diversity	3.23	3.05	3.13	0.155	0.73	0.67	0.52
Inverse Simpson’s diversity	10.0	8.3	9.3	1.30	0.65	0.72	0.41
Day 15							
Number of OTUs	235.0	223.5	213.3	28.60	0.87	0.60	0.99
Shannon diversity	3.21	3.09	3.08	0.115	0.69	0.45	0.71
Inverse Simpson’s diversity	9.4	8.8	8.5	0.53	0.51	0.27	0.86


### Effect of Biochar on Rumen Fungal Microbiota

**FIGURE 3:**
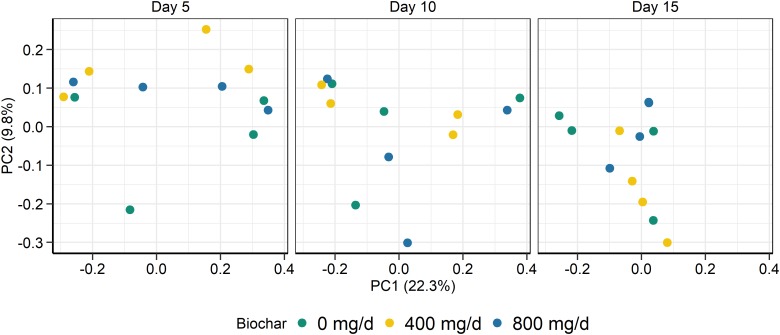
Principal coordinates plot (PCoA) of the Bray-Curtis dissimilarities for the fungal solid-associated microbe (SAM) samples by sampling time and biochar concentration. The percentage of variation explained by each principal coordinate are indicated on the axes.

**FIGURE 4:**
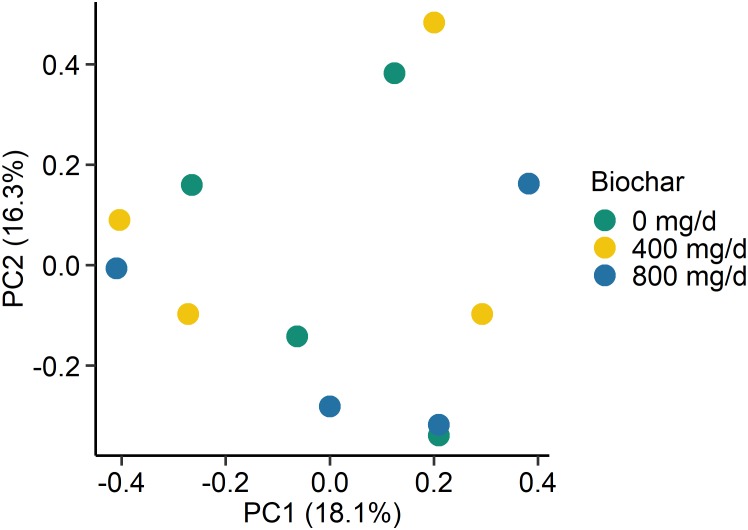
Principal coordinates plot (PCoA) of the Bray-Curtis dissimilarities for the fungal liquid-associated microbe (LAM) samples by biochar concentration. The percentage of variation explained by each principal coordinate are indicated on the axes.

As with the archaeal and bacterial rumen microbiota, the fungal community structure was not affected by biochar addition for neither SAM (*P* > 0.05; Figure 3) nor LAM samples (*P* > 0.05; Figure 4). Although no fungal OTUs were differentially abundant between the 0 mg/g vs. 800 mg/d biochar treatments in the SAM samples at d 15 (*P* > 0.05), there were two OTUs that had significantly different abundances between these treatments in the LAM samples (*P* < 0.05). These included one OTU classified as *Vishniacozyma victoriae* (5.4 × 10^7^-fold increase) and another as *Sporobolomyces ruberrimus* (5.4 × 10^7^-fold increase), both more abundant in the 800 mg/d biochar samples. *Aspergillus intermedius, Monascus purpureus*, and *Vishniacozyma victoriae* were relatively abundant (>3.0%) in both the SAM and LAM samples (Supplementary Figures S3, S4), otherwise there was more variability among the samples than with the archaeal and bacterial microbiota. Biochar addition also did not affect the fungal richness and diversity for both LAM and SAM samples (Table 5; *P* > 0.05).

**Table 5:** Effect of biochar on archaeal and bacterial richness and diversity in a RUSITEC system, with a mixed hay-silage-concentrate diet.

	Biochar (mg/d)				*P*-value		
			
	Control, 0	400	800	SEM	Treat	L	Q
**Liquid-associated microbes**							
Day 15							
Number of OTUs	25.5	19.3	19.5	8.55	0.80	0.61	0.75
Shannon diversity	2.3	1.9	1.9	0.43	0.45	0.31	0.47
Inverse Simpson’s diversity	9.0	4.8	6.2	2.74	0.31	0.31	0.25
**Solid-associated microbes**							
Day 5							
Number of OTUs	47.0	39.0	43.0	6.66	0.71	0.68	0.48
Shannon diversity	2.8	2.7	2.7	0.14	0.82	0.76	0.60
Inverse Simpson’s diversity	9.8	8.5	8.6	1.11	0.69	0.49	0.66
Day 10							
Number of OTUs	49.8	40.8	38.5	6.11	0.48	0.28	0.68
Shannon diversity	2.9	2.8	2.9	0.13	0.78	0.83	0.54
Inverse Simpson’s diversity	10.0	10.6	13.3	1.68	0.37	0.20	0.61
Day 15							
Number of OTUs	54.8	47.8	45.3	6.53	0.59	0.33	0.78
Shannon diversity	3.0	3.1	2.8	0.15	0.49	0.44	0.38
Inverse Simpson’s diversity	12.2	14.5	10.8	2.42	0.61	0.70	0.39


*SEM, standard error of the means; L, linear; Q, quadratic.*

## Discussion

Biochar supplementation had no effect on pH, effluent or total gas production during rumination. Similar results were reproduced in another RUSITEC system by Saleem et al. (2018). They were unable to detect a significant relationship between the addition of pine biochar and pH, total gas production, and protozoa numbers. A common feature shared between this study and Saleem et al. (2018) was the use of relatively high-quality forage. McFarlane et al. (2017) proposed that biochar selectively improved fermentation kinetics when incubated with forage of intermediate quality, in contrast to higher-quality materials. This statement is supported by the absence of biochar effect on several fermentation characteristics in this study and Saleem et al. (2018). In both studies, base substrates held a greater CP (10.54 and 16.1% vs. 9.83%) and lower NDF (45.79 and 36.9% vs. 70.4%) compared to McFarlane et al. (2017). Forage quality is known to alter rumen microbiota *in vitro* (Iqbal et al., 2018), and therefore influence VFA concentration and other fermentation parameters *in vivo* (Calsamiglia et al., 2008; Pino et al., 2018). However, further studies are required to examine the effect of a single biochar treatment on various forage compositions, and whether there is a more complex causal relationship, before conclusions on this theory can be made.

There was no significant difference in CH_4_ production between diets with a hardwood biochar additive, and those without. This absence of effect on CH_4_ production has been replicated *in vitro* by Calvelo Pereira et al. (2014), despite utilizing different biomass sources (i.e., corn stover and pine wood chips), biochar pyrolysis temperatures (i.e., 350 and 550°C) and biochar dose rates (i.e., 0, 2.1, 4.2, 8.1 and 18.6% substrate DM). However, several publications have suggested the contrary, exhibiting biochar’s ability to successfully reduce CH_4_ production (Hansen et al., 2012; Leng et al., 2012a; McFarlane et al., 2017; Cabeza et al., 2018; Saleem et al., 2018). Hansen et al. (2012) reported an tendency *in vitro* for CH_4_ emissions to be reduced by 11 to 17% across 4 different biocarbon sources (i.e., wood, gas, straw and activated charcoal), while Leng et al. (2012c) demonstrated an enteric CH_4_ production drop of 20% with rice husk biochar. The variable success rate of past biochar studies at reproducing significant CH_4_ mitigation results has largely been attributed to variation in biochar properties. Namely, the particle size, adsorptive potential, electrical conductivity, and ability to act as an electron mediator in redox reactions during digestion (Hansen et al., 2012; Olivier, 2013; Klupfel et al., 2014; Yu et al., 2015; Kammann et al., 2017; Leng, 2017; McFarlane et al., 2017) as well as directly affecting the bacterial biofilm and/or individual microbial populations. The addition of biochar as an electron mediator has been proposed to increase efficiency of redox reactions via electron transfers directly between ruminal microbial species (Chen et al., 2010; Kammann et al., 2017). This would ultimately improve feed-conversion efficiency and reduce GHG emissions – a form of energy wastage (Leng et al., 2012a,c; Kammann et al., 2017).

The difference in biochar pH may provide a possible explanation as to why the mineral-activated biochar used in this study had no effect on CH_4_ production. The biochar compounds used in this study, along with those used by Hansen et al. (2012), Calvelo Pereira et al. (2014) were relatively basic (pH 8.2, 9.8, and Hansen et al. (2012) pH 10.2 and 9.6 respectively), and showed no significant effect on *in vitro* CH_4_ production. In contrast, Saleem et al. (2018) used acidic (pH 4.8) biochar which reduced CH_4_ emissions. It is known that redox potential and pH are major drivers of microbial systems (Olivier, 2013), with changes in pH negatively correlated with ruminal redox potential (Huang et al., 2016, 2018). Transient pH variations are also known to cause long-term impacts on the ruminal microbiota (Castillo-González et al., 2014; Eger et al., 2018). Carbon-rich acidic biochar has been associated with heightened carbon sequestrum in soils, whereas neutral mineral-rich biochar lacked this ability (Qi et al., 2017). Biochar’s electron-donating capacity has also been suggested to affect methanogenesis in food waste (Cruz Viggi et al., 2017). Therefore, this suggests that acidic carbon-rich biochar holds a higher ruminal redox potential and is more effective at driving rumen fermentation toward a more efficient energy conversion spectrum by manipulating microbial populations. To confirm this, future studies should be directed at characterizing and comparing the rumen microbial community post-supplementation, using biochars of differing pH values, electron-donating capacities and mineral or carbon composition.

The addition of hardwood biochar at 800 mg/d (7.2% DM) tended to decrease the % of CH_4_ in the gas sample during fermentation, in contrast to a biochar dose of 400 mg/d (3.6% DM). This suggests a possible dose-responsive relationship, where in this study a minimum of 7.2% DM of hardwood biochar additive was required before a potential effect may be seen. Leng et al. (2012b) also suggested that biochar may have an additive effect during *in vitro* incubation. Greater methane mitigation was seen when biochar supplement was added to ruminal fluid collected from cattle already adapted to 0.62% biochar over 4 months, compared to incubation groups that either had not received biochar or the adapted rumen fluid (Leng et al., 2012b). Similarly Saleem et al. (2018) exhibited a dose-dependent relationship between reducing CH_4_ production and increasing doses of dietary biochar in a quadratic fashion. Yet there is conflicting evidence suggesting that there is no effect on fermentation irrespective of biochar dosage (Cabeza et al., 2018). This disparity can potentially be attributed to the use of 24-h batch cultures, an *in vitro* method that is much simpler than the complex continuous fermentation reactions seen in RUSITEC and *in vivo* studies. Another avenue to consider is rather than an absolute drop in % CH_4_ produced, it is possible that biochar instead caused an increase in the production of a different type of gas that was not measured in this study, e.g., NH_3_, CO_2_. Theoretically, this would result in what seems like an overall “drop” in CH_4_ proportion. It should also be noted that in a previous study (Saleem et al., 2018), only 0.5% DM biochar supplement was required for a significant effect, compared to the 7.2% DM needed for a statistical tendency in our study. The large discrepancy between levels of biochar supplementation may limit the practical implementation of this supplement. Excessive supplementation of hardwood biochar can limit energy intake in cattle via energy-dilution of the diet while reducing feed palatability substantially (Erickson et al., 2011; Cabeza et al., 2018). Therefore, hardwood biochar’s practical feasibility remains in question.

No effect on DM digestibility was seen with biochar supplementation in the present study. Biochar is 100% inorganic matter and not metabolized by the rumen microbiome. This result is in disagreement with Saleem et al. (2018), where they elicited a positive response in DM digestibility using pine-sourced biochar in a similar RUSITEC system. Erickson et al. (2011) also showed *in vivo* improvements in nutrient digestibility in mycotoxin-laden silage post-biochar addition. Although there is evidence suggesting that biochar has no effect on digestibility (Hansen et al., 2012; McFarlane et al., 2017), these studies were performed using batch cultures which can only partially imitate natural rumination. Furthermore, differences in methodology and biochar qualities can affect the formation of fermentative and detoxifying biofilms (Leng, 2014, 2017). While the rumen microbiota carry genes responsible for the metabolism of harmful diet constituents, these are minimally expressed in the normal state due to amensalism (Leng, 2014, 2017). The addition of biochar during incubation encourages biofilm creation, which in turn stimulates the growth of these desirable microbes by providing a niche for their continued proliferation (Leng, 2014, 2017). Efforts have been made to characterize these affected microbial communities in the present study and to the best of our knowledge, this is the first investigation of the impact of biochar on the ruminal microbiota.

Biochar supplementation had no effect on total and individual VFA production throughout the *in vitro* fermentation. This is in stark contrast to previous studies which indicated that either total VFA production, individual VFAs (i.e., acetate, propionate, butyrate, branch-chained VFAs) or both were significantly increased with biochar supplementation (Calvelo Pereira et al., 2014; McFarlane et al., 2017; Cabeza et al., 2018; Saleem et al., 2018). However, the lack of VFA response in our study corresponds to the absence of any significant biochar effect on the rumen archaeal, bacterial and fungal microbiota structure. The most relatively abundant bacterial taxa measured in both SAM and LAM samples were unaffected by biochar, and the microbiota of both sample types was dominated by *Megasphaera* and *Prevotella* spp., which are members of the Firmicutes and Bacteroidetes phyla, respectively. Similar core bacterial groups have already been characterized in dairy cattle and other ruminants (de Menezes et al., 2011; Henderson et al., 2015; Derakhshani et al., 2016; Duarte et al., 2017; Zuo et al., 2017). A study of biochar supplementation in poultry diets at a concentration of 4% w/w was also reported to have no effect on cloacal microbial richness and diversity (Prasai et al., 2016).

Despite no significant biochar effect on microbial diversity and community structure, there was a 19.8-fold reduction in the abundance of an OTU classified as Methanomethylophilaceae and a 31.7-fold decrease in a *Lactobacillus* OTU in the 800 mg/d biochar SAM samples. *Lactobacillus* spp. have long since been characterized within the rumen microbiota, and have a major role in the production of lactic acid, CO_2_ and acetic acid via carbohydrate fermentation (Edmondson et al., 1956; Marounek et al., 1988; Hernandez et al., 2008). Methanomethylophilaceae, on the other hand, is a relatively new evolutionary family clade that is almost exclusively adapted for the gastrointestinal environment and is within the Methanomassiliicoccales order (previously referred to as Methanoplasmatales) (Borrel et al., 2014; Gaci et al., 2014). This methanogenic family relies on an external source of hydrogen for the reduction of methylated compounds to produce methane (Borrel et al., 2014; Brugere et al., 2014; Lang et al., 2015). A wide variation in Methanomassiliicoccales abundance has been described across many ruminant species given varied diets and based in different geographical regions, ranging from 0.5% (Jin et al., 2017) to more than 50% of the total rumen archaea (St-Pierre and Wright, 2013; Seedorf et al., 2015). The decrease in Methanomethylophilaceae abundance suggests that hardwood biochar had an inhibitory effect on this family. It is currently unknown whether the reduction in the abundance of Methanomethylophilaceae members is also the reason for ruminal methane reductions in other biochar supplementation studies.

Biochar supplementation at 800 mg/d also significantly increased the abundance of two OTUs classified as *Vishniacozyma victoriae* (5.4 × 10^7^-fold increase, phylum Ascomycota) and *Sporobolomyces ruberrimus* (5.4 × 10^7^-fold increase). *V. victoriae* (also known as *Cryptococcus victoriae*) and *S. ruberrimus* are both species of yeast; *V. victoriae* has been identified in cow’s milk (Delavenne et al., 2011) and meat processing plants (Nielsen et al., 2008) and *S. ruberrimus* in chicken ceca (Byrd et al., 2017).

There are several superficial conflicts in our study’s results. Biochar supplementation caused significant changes in several OTU abundances across bacterial, fungal and archaeal populations. It is also important to note that methanogen abundance does not always translate directly to CH_4_ production (Hook et al., 2009; Popova et al., 2011; Singh et al., 2013; Scharen et al., 2017). Instead, other unidentified relationships between individualized host genetics and microbiome biochemical pathways also contribute in determining CH_4_ production (Zhou et al., 2009, 2010; Roehe et al., 2016; Malmuthuge and Guan, 2017; Schären et al., 2018). This may help explain the logical friction between the drop in Methanomethylophilaceae abundance, and the limited effect on absolute CH_4_ produced in this study.

The practical use of biochar is currently constrained by conflicting study results brought about by the substantial differences in methodology utilized. Variations in biochar manufacturing and regionality have been shown to affect end-product characteristics and adsorptive ability (Lehmann and Joseph, 2015; McFarlane et al., 2017; Cabeza et al., 2018). While this capacity holds promising future potential for personalized production, it presents a roadblock in terms of study comparison and analysis to make meaningful conclusions on biochar’s effects. Specifically, there is substantial diversity in the pyrolysis temperatures and biomass source amongst studies. This in turn bears complications for result interpretation. For example, variations of 350°C and 550°C, 700°C, and 900°C in Leng et al. (2012a), Calvelo Pereira et al. (2014), and Cabeza et al. (2018), respectively. Some of the studied biomass sources include chestnut oak, yellow poplar and white pine (McFarlane et al., 2017), corn stover and pine wood chips (Calvelo Pereira et al., 2014), *Miscanthus* straw, oil seed rape straw, rice husk, soft wood pellets and wheat straw (Cabeza et al., 2018). However, evidence exists that suggests lower pyrolysis temperatures encourage total gas, CH_4_, ammonia (NH_3_) and VFA production (Calvelo Pereira et al., 2014; Cabeza et al., 2018), while biomass source can affect total VFA, NH_3_ and individual amounts of acetic and butyric acid concentrations (Cabeza et al., 2018). Therefore, the quality of biochar has a significant impact on fermentation characteristics and can introduce confounding factors into literature comparison.

In addition, the biochar used in many studies is poorly characterized. Despite particle size having recently been put forward as having an influence on the physicochemical properties of biochar (Zhou et al., 2017; He et al., 2018), and the determination of CH_4_ production and rumen fermentation (McFarlane et al., 2017), it is impossible to draw comparisons between previous studies as this information has not been included. Even our study has no comparable counterpart published at this time, as an *in vitro* RUSITEC study utilizing a novel biochar incorporating minerals bentonite and zeolite. Bentonite has previously been shown to have a tendency to reduce methane production (Leng et al., 2013). It has also been reported to absorb and improve the conversion efficiency of NH_3_ in microbial protein synthesis, increase live weight gain and activity of ruminal protozoa in Holstein bulls and dairy calves (Kirovski et al., 2015; Mohsen et al., 2017). Zeolite has been used to improve milk yield and increase acetate:propionate ratio in lactating cows (Khachlouf et al., 2018), and elevate total VFA and organic acid rumen concentration in lambs (Erwanto et al., 2012). Yet these beneficial qualities are either unable to be replicated or remain to be seen in the hardwood biochar end-product used in this study. This suggests that the qualities of mineral additives cannot survive pyrolysis processing and are perhaps more useful as unprocessed supplements.

## Conclusion

Biochar supplementation at 800 mg/d (7.2% DM) tended to reduce methane concentration compared to 400 mg/d (3.6% DM). In contrast, hardwood biochar had no effect on pH, effluent volume, DM digestibility, VFA, CH_4_, or total gas production. Additionally, there was a suppressive effect on ruminal OTUs identified as Methanomethylophilaceae and *Lactobacillus* spp., a positive effect on OTUs classified as *Vishniacozyma victoriae* and *Sporobolomyces ruberrimus.* There is currently no standardized method or baseline on which biochar characteristics should be reported in a study. Consequently, comparative analysis of past literature and identification of the biological mechanisms responsible for the results found in this study is challenging. Future biochar studies with characterisation of pH, particle size, adsorptive potential, electron-donating capacity, electrical conductivity and redox potential would be ideal to investigate this relationship.

## Data Availability

The datasets generated for this study can be found in all 16S rRNA genes and ITS sequences were submitted to the Sequence Read Archive under the BioProject, PRJNA525436.

## Ethics Statement

The donor cow was cared for in accordance with The University of Sydney Animal Ethics Committee (Project Number 2015/835) and housed at The University of Sydney, Corstorphine (Camden Farm Dairy, Cobbitty, NSW, Australia).

## Author Contributions

AC designed the study, acquired the data, conducted laboratory and statistical analyses, and wrote the manuscript. RT acquired the data, conducted laboratory analyses, and wrote the manuscript. EC acquired the data and conducted laboratory analyses. SJ conducted laboratory analyses. DH conducted bioinformatics, statistical analysis, and wrote the manuscript. SM wrote the manuscript. All authors read the final manuscript, critically revised it for intellectual contents and approved it for publication.

## Conflict of Interest Statement

The authors declare that the research was conducted in the absence of any commercial or financial relationships that could be construed as a potential conflict of interest.

## Supplementary Material

FIGURE S1 Box and whisker plots of the 10 most relative abundant bacterial genera in the solid-associated microbe (SAM) rumen samples by sampling time and biochar concentration. The box in the box plots indicates the interquartile range (IQR) (middle 50% of the data), the middle line represents the median value, and the whiskers represents 1.5 times the IQR.

FIGURE S2 Box and whisker plots of the 10 most relative abundant bacterial genera in the liquid-associated microbe (LAM) rumen samples by sampling time and biochar concentration. The box in the box plots indicates the interquartile range (IQR) (middle 50% of the data), the middle line represents the median value, and the whiskers represents 1.5 times the IQR. Different lowercase letters indicate significantly different means (*P* ≤ 0.05).

FIGURE S3 Box and whisker plots of the 10 most relatively abundant fungal species in the solid-associated microbe (SAM) rumen samples by sampling time and biochar concentration. The box in the box plots indicates the interquartile range (IQR) (middle 50% of the data), the middle line represents the median value, and the whiskers represents 1.5 times the IQR. Different lowercase letters within each sampling time represent significantly different means (*P* < 0.05).

FIGURE S4 Box and whisker plots of the 10 most relative abundant fungal species in the liquid-associated microbe (LAM) rumen samples by sampling time and biochar concentration. The box in the box plots indicates the interquartile range (IQR) (middle 50% of the data), the middle line represents the median value, and the whiskers represents 1.5 times the IQR.

## References

[B1] Agegnehu G. Srivastava A. K. Bird M. I. (2017). The role of biochar and biochar-compost in improving soil quality and crop performance: a review. *Appl. Soil Ecol.* 119 156–170. 10.1016/j.apsoil.2017.06.008

[B2] Association of Official Analytical Chemists [AOAC] (2006). *Official Methods of Analysis.* Gaithersburg: AOAC International.

[B3] Beauchemin K. Mo K. O’mara F. Mcallister T. (2008). Nutritional management for enteric methane abatement: a review. *Aust. J. Exp. Agric.* 48 21–27.

[B4] Borrel G. Parisot N. Harris H. M. Peyretaillade E. Gaci N. Tottey W. (2014). Comparative genomics highlights the unique biology of Methanomassiliicoccales, a Thermoplasmatales-related seventh order of methanogenic archaea that encodes pyrrolysine. *BMC Genomics* 15:679. 10.1186/1471-2164-15-679 25124552 PMC4153887

[B5] Broucek J. (2014). Production of methane emissions from ruminant husbandry: a review. *J. Environ. Protect.* 5 1482–1493. 10.4236/jep.2014.515141

[B6] Brugere J. F. Borrel G. Gaci N. Tottey W. O’toole P. W. Malpuech-Brugere C. (2014). Archaebiotics: proposed therapeutic use of archaea to prevent trimethylaminuria and cardiovascular disease. *Gut Microbes* 5 5–10. 10.4161/gmic.26749 24247281 PMC4049937

[B7] Buee M. Reich M. Murat C. Morin E. Nilsson R. H. Uroz S. (2009). 454 Pyrosequencing analyses of forest soils reveal an unexpectedly high fungal diversity. *New Phytol.* 184 449–456. 10.1111/j.1469-8137.2009.03003.x 19703112

[B8] Byrd J. A. Caldwell D. Y. Nisbet D. J. (2017). The identification of fungi collected from the ceca of commercial poultry. *Poult. Sci.* 96 2360–2365. 10.3382/ps/pew486 28339796

[B9] Cabeza I. Waterhouse T. Sohi S. Rooke J. A. (2018). Effect of biochar produced from different biomass sources and at different process temperatures on methane production and ammonia concentrations in vitro. *Anim. Feed Sci. Technol.* 237 1–7. 10.1016/j.anifeedsci.2018.01.003

[B10] Callahan B. J. Mcmurdie P. J. Rosen M. J. Han A. W. Johnson A. J. Holmes S. P. (2016). DADA2: high-resolution sample inference from Illumina amplicon data. *Nat. Methods* 13 581–583. 10.1038/nmeth.3869 27214047 PMC4927377

[B11] Calsamiglia S. Cardozo P. W. Ferret A. Bach A. (2008). Changes in rumen microbial fermentation are due to a combined effect of type of diet and pH. *J. Anim. Sci.* 86 702–711. 10.2527/jas.2007-0146 18073289

[B12] Calvelo Pereira R. Muetzel S. Camps Arbestain M. Bishop P. Hina K. Hedley M. (2014). Assessment of the influence of biochar on rumen and silage fermentation: a laboratory-scale experiment. *Anim. Feed Sci. Technol.* 196 22–31. 10.1016/j.anifeedsci.2014.06.019

[B13] Castillo-González A. R. Burrola-Barraza M. E. Domínguez-Viverosb J. Chávez-Martínez A. (2014). Rumen microorganisms and fermentation. *Arch. Med. Vet.* 46 349–361. 10.4067/s0301-732x2014000300003

[B14] Chagunda M. G. G. Römer D. A. M. Roberts D. J. (2009). Effect of genotype and feeding regime on enteric methane, non-milk nitrogen and performance of dairy cows during the winter feeding period. *Livest. Sci.* 122 323–332. 10.1016/j.livsci.2008.09.020

[B15] Chen Y.-X. Huang X.-D. Han Z.-Y. Huang X. Hu B. Shi D.-Z. (2010). Effects of bamboo charcoal and bamboo vinegar on nitrogen conservation and heavy metals immobility during pig manure composting. *Chemosphere* 78 1177–1181. 10.1016/j.chemosphere.2009.12.029 20060567

[B16] Cruz Viggi C. Simonetti S. Palma E. Pagliaccia P. Braguglia C. Fazi S. (2017). Enhancing methane production from food waste fermentate using biochar: the added value of electrochemical testing in pre-selecting the most effective type of biochar. *Biotechnol. Biofuels* 10:303. 10.1186/s13068-017-0994-7 29255486 PMC5729428

[B17] de Menezes A. B. Lewis E. O’donovan M. O’neill B. F. Clipson N. Doyle E. M. (2011). Microbiome analysis of dairy cows fed pasture or total mixed ration diets. *FEMS Microbiol. Ecol.* 78 256–265. 10.1111/j.1574-6941.2011.01151.x 21671962

[B18] Delavenne E. Mounier J. Asmani K. Jany J. L. Barbier G. Le Blay G. (2011). Fungal diversity in cow, goat and ewe milk. *Int. J. Food Microbiol.* 151 247–251. 10.1016/j.ijfoodmicro.2011.08.029 21944758

[B19] Deppenmeier U. (2002). The unique biochemistry of methanogenesis. *Prog. Nucleic Acid Res. Mol. Biol.* 71 223–283. 10.1016/s0079-6603(02)71045-3 12102556

[B20] Derakhshani H. Tun H. M. Li S. Plaizier J. C. Khafipour E. Moossavi S. (2016). Effects of grain feeding on microbiota in the digestive tract of cattle. *Anim. Front.* 6 13–19. 10.2527/af.2016-0018

[B21] Duarte A. C. Holman D. B. Alexander T. W. Durmic Z. Vercoe P. E. Chaves A. V. (2017). The type of forage substrate preparation included as substrate in a RUSITEC system affects the ruminal microbiota and fermentation characteristics. *Front. Microbiol.* 8:704. 10.3389/fmicb.2017.00704 28473826 PMC5397515

[B22] Edmondson J. E. Jensen R. G. Merilan C. P. Smith K. L. (1956). The characteristics of some rumen lactobacilli. *J. Bacteriol.* 72 253–258. 13366908 10.1128/jb.72.2.253-258.1956 PMC357888

[B23] Eger M. Riede S. Breves G. (2018). Induction of a transient acidosis in the rumen simulation technique. *J. Anim. Physiol. Anim. Nutr.* 102 94–102. 10.1111/jpn.12662 28299854

[B24] Erickson P. S. Whitehouse N. L. Dunn M. L. (2011). Activated carbon supplementation of dairy cow diets: effects on apparent total-tract nutrient digestibility and taste preference 1: 1. *Prof. Anim. Sci.* 27:428 10.15232/s1080-7446(15)30515-5

[B25] Erwanto E. Zakaria W. A. Prayuwidayati M. (2012). The use of ammoniated zeolite to improve rumen metabolism in ruminant. *Anim. Prod.* 13 138–142.

[B26] Food and Agriculture Organization of the United Nations (2008). “The State of Food Insecurity in the World 2008. High food prices and food security - threats and opportunities,” in *The State of the World*, (Rome: Food and Agriculture Organization of the United Nations). Available at: http://www.fao.org/3/i0291e/i0291e00.htm

[B27] Food and Agriculture Organization of the United Nations, International Fund for Agricultural Development, United Nations Children’s Fund, World Food Programme, and World Health Organization (2018). “The State of Food Security and Nutrition in the World 2018. Building climate resilience for food security and nutrition,” in *The State of the World*, (Rome: Food and Agriculture Organization of the United Nations).

[B28] Gaci N. Borrel G. Tottey W. O’toole P. W. Brugere J. F. (2014). Archaea and the human gut: new beginning of an old story. *World J. Gastroenterol.* 20 16062–16078. 10.3748/wjg.v20.i43.16062 25473158 PMC4239492

[B29] Gerber P. J. (2013). *Tackling Climate Change Through Livestock: A Global Assessment of Emissions and Mitigation Opportunities.* Rome: Food and Agriculture Organization of the United Nations.

[B30] Hansen H. H. Storm I. M. L. D. Sell A. M. (2012). Effect of biochar on in vitro rumen methane production. *Acta Agric. Scand., Sec. A Anim. Sci.* 62 305–309. 10.1093/jas/sky204 29912357 PMC6095387

[B31] Haque M. N. (2018). Dietary manipulation: a sustainable way to mitigate methane emissions from ruminants. *J. Anim. Sci. Technol.* 60:15. 10.1186/s40781-018-0175-7 29946475 PMC6004689

[B32] He P. Liu Y. Shao L. Zhang H. Lü F. (2018). Particle size dependence of the physicochemical properties of biochar. *Chemosphere* 212 385–392. 10.1016/j.chemosphere.2018.08.106 30149311

[B33] Henderson G. Cox F. Ganesh S. Jonker A. Young W. Abecia L. (2015). Rumen microbial community composition varies with diet and host, but a core microbiome is found across a wide geographical range. *Sci. Rep.* 5:23. 10.1016/j.mib.2016.11.002 26449758 PMC4598811

[B34] Hernandez J. D. Scott P. T. Shephard R. W. Al Jassim R. A. (2008). The characterization of lactic acid producing bacteria from the rumen of dairy cattle grazing on improved pasture supplemented with wheat and barley grain. *J. Appl. Microbiol.* 104 1754–1763. 10.1111/j.1365-2672.2007.03696.x 18217928

[B35] Hook S. E. Northwood K. S. Wright A.-D. G. Mcbride B. W. (2009). Long-term monensin supplementation does not significantly affect the quantity or diversity of methanogens in the rumen of the lactating dairy cow. *Appl. Environ. Microbiol.* 75 374–380. 10.1128/AEM.01672-08 19028912 PMC2620707

[B36] Hristov A. N. Oh J. Lee C.-C. Meinen R. Montes F. Ott T. (2013). “Mitigation of greenhouse gas emissions in livestock production - a review of technical options for non-CO2 emissions,” in *FAO Animal Production and Health Paper*, eds Gerber P. J. Henderson B. Makkar H. P. S. (Rome: FAO). 10.1017/S1751731113000876 23739465

[B37] Huang Y. Julien C. Philippe Marden J. Bayourthe C. (2016). “Relationship between ruminal redox potential and pH in dairy cattle,” in *Proceedings of the 20th Congress of the European Society of Veterinary and Comparative Nutrition (ESVCN)*, Berlin.

[B38] Huang Y. Marden J. P. Julien C. Bayourthe C. (2018). Redox potential: an intrinsic parameter of the rumen environment. *J. Anim. Physiol. Anim. Nutr.* 102 393–402. 10.1111/jpn.12855 29352497

[B39] Iqbal M. W. Zhang Q. Yang Y. Zou C. Li L. Liang X. (2018). Ruminal fermentation and microbial community differently influenced by four typical subtropical forages in vitro. *Anim. Nutr.* 4 100–108. 10.1016/j.aninu.2017.10.005 30167491 PMC6112341

[B40] Jeffery S. Verheijen F. G. A. Kammann C. Abalos D. (2016). Biochar effects on methane emissions from soils: a meta-analysis. *Soil Biol. Biochem.* 101 251–258. 10.1016/j.soilbio.2016.07.021

[B41] Jin W. Cheng Y. Zhu W. (2017). The community structure of Methanomassiliicoccales in the rumen of Chinese goats and its response to a high-grain diet. *J. Anim. Sci. Biotechnol.* 8 47–47. 10.1186/s40104-017-0178-0 28572975 PMC5452365

[B42] Joseph S. Pow D. Dawson K. Mitchell D. R. G. Rawal A. Hook J. (2015). Feeding biochar to cows: an innovative solution for improving soil fertility and farm productivity. *Pedosphere* 25 666–679. 10.1016/s1002-0160(15)30047-3

[B43] Kammann C. Ippolito J. Hagemann N. Borchard N. Cayuela M. L. Estavillo J. M. (2017). Biochar as a tool to reduce the agricultural greenhouse-gas burden – knowns, unknowns and future research needs. *J. Environ. Eng. Landsc. Manag.* 25 114–139. 10.3846/16486897.2017.1319375

[B44] Khachlouf K. Hamed H. Gdoura R. Gargouri A. (2018). Effects of zeolite supplementation on dairy cow production and ruminal parameters - a review. *Ann. Anim. Sci.* 18 1–21.

[B45] Kirovski D. Adamovic M. Radivojevic M. Samanc H. Vujanac I. Prodanovic R. (2015). Effects of bentonite on weight gain, feed consumption, blood metabolites and ruminal protozoa in dairy calves. *Anim. Nutr. Feed Technol.* 15 11–20.

[B46] Klupfel L. Keiluweit M. Kleber M. Sander M. (2014). Redox properties of plant biomass-derived black carbon (biochar). *Environ. Sci. Technol.* 48 5601–5611. 10.1021/es500906d 24749810

[B47] Koljalg U. Nilsson R. H. Abarenkov K. Tedersoo L. Taylor A. F. Bahram M. (2013). Towards a unified paradigm for sequence-based identification of fungi. *Mol. Ecol.* 22 5271–5277. 10.1111/mec.12481 24112409

[B48] Lang K. Schuldes J. Klingl A. Poehlein A. Daniel R. Brune A. (2015). New mode of energy metabolism in the seventh order of methanogens as revealed by comparative genome analysis of “candidatus methanoplasma termitum”. *Appl. Environ. Microbiol.* 81 1338–1352. 10.1128/aem.03389-14 25501486 PMC4309702

[B49] Lehmann J. Joseph S. (2015). *Biochar for Environmental Management: Science, Technology and Implementation.* London: Taylor and Francis Ltd.

[B50] Leng R. A. (2014). Interactions between microbial consortia in biofilms: a paradigm shift in rumen microbial ecology and enteric methane mitigation. *Anim. Prod. Sci.* 54 519–543.

[B51] Leng R. A. (2017). “Biochar in Ruminant Nutrition and Health,” in *Proceedings of the Australia New Zealand Biochar Conference*, (Wagga Wagga NSW: AgriFutures Australia), 12–13.

[B52] Leng R. A. Inthapanya S. Preston T. (2012a). Biochar lowers net methane production from rumen fluid in vitro. *Livest. Res. Rural Dev.* 24:103.

[B53] Leng R. A. Inthapanya S. Preston T. (2012b). Methane production is reduced in an in vitro incubation when the rumen fluid is taken from cattle that previously received biochar in their diet. *Livest. Res. Rural Dev.* 24:211.

[B54] Leng R. A. Preston T. Inthapanya S. (2012c). Biochar reduces enteric methane and improves growth and feed conversion in local “Yellow” cattle fed cassava root chips and fresh cassava foliage. *Livest. Res. Rural Dev.* 24:199.

[B55] Leng R. A. Inthapanya S. Preston T. (2013). All biochars are not equal in lowering methane production in in vitro rumen incubations. *Livest. Res. Rural Dev.* 25:106.

[B56] Love M. I. Huber W. Anders S. (2014). Moderated estimation of fold change and dispersion for RNA-seq data with DESeq2. *Genome Biol.* 15:550. 25516281 10.1186/s13059-014-0550-8 PMC4302049

[B57] Malmuthuge N. Guan L. L. (2017). Understanding host-microbial interactions in rumen: searching the best opportunity for microbiota manipulation. *J. Anim. Sci. Biotechnol.* 8:8. 10.1186/s40104-016-0135-3 28116074 PMC5244612

[B58] Marounek M. Jehlickova K. Kmet V. (1988). Metabolism and some characteristics of lactobacilli isolated from the rumen of young calves. *J. Appl. Bacteriol.* 65 43–47. 10.1111/j.1365-2672.1988.tb04315.x 3209515

[B59] McFarlane Z. Myer P. Cope E. Evans N. D. Bone C. Biss B. (2017). Effect of biochar type and size on in vitro rumen fermentation of orchard grass hay. *Agric. Sci.* 8 316–325. 10.4236/as.2017.84023

[B60] McMurdie P. J. Holmes S. (2013). phyloseq: an R package for reproducible interactive analysis and graphics of microbiome census data. *PLoS One* 8:e61217. 10.1371/journal.pone.0061217 23630581 PMC3632530

[B61] Mertens D. R. (1997). Creating a system for meeting the fiber requirements of dairy cows. *J. Dairy Sci.* 80 1463–1481. 10.3168/jds.S0022-0302(97)76075-2 9241608

[B62] Mohsen K. Sirjani M. K. Tahmasbi A. M. Khoram A. E. I. Torbaghan A. E. (2017). Effects of sodium and calcium bentonite on growth performance and rumen ammonia in Holstein bulls. *Livest. Res. Rural Dev.* 29:144.

[B63] Nielsen D. S. Jacobsen T. Jespersen L. Koch A. G. Arneborg N. (2008). Occurrence and growth of yeasts in processed meat products - Implications for potential spoilage. *Meat Sci.* 80 919–926. 10.1016/j.meatsci.2008.04.011 22063618

[B64] Oksanen J. Blanche F. G. Kindt R. Legendre P. Minchin P. R. O’hara R. (2013). *Package ‘Vegan’. Community Ecology Package, Version* 2.

[B65] Olivier H. (2013). Redox potential (Eh) and pH as drivers of soil/plant/microorganism systems: a transdisciplinary overview pointing to integrative opportunities for agronomy. *Plant Soil* 362 389–417. 10.1007/s11104-012-1429-7

[B66] Pino F. Mitchell L. K. Jones C. M. Heinrichs A. J. (2018). Comparison of diet digestibility, rumen fermentation, rumen rate of passage, and feed efficiency in dairy heifers fed ad-libitum versus precision diets with low and high quality forages. *J. Appl. Anim. Res.* 46 1296–1306. 10.1080/09712119.2018.1498788

[B67] Popova M. Martin C. Eugène M. Mialon M. M. Doreau M. Morgavi D. P. (2011). Effect of fibre- and starch-rich finishing diets on methanogenic Archaea diversity and activity in the rumen of feedlot bulls. *Anim. Feed Sci. Technol.* 16 113–121.

[B68] Prasai T. P. Walsh K. B. Bhattarai S. P. Midmore D. J. Van T. T. H. Moore R. J. (2016). Biochar, bentonite and zeolite supplemented feeding of layer chickens alters intestinal microbiota and reduces *campylobacter* load. *PLoS One* 11:e0154061. 10.1371/journal.pone.0154061 27116607 PMC4845986

[B69] Qi F. Dong Z. Lamb D. Naidu R. Bolan N. S. Ok Y. S. (2017). Effects of acidic and neutral biochars on properties and cadmium retention of soils. *Chemosphere* 180 564–573. 10.1016/j.chemosphere.2017.04.014 28437653

[B70] Quast C. Pruesse E. Yilmaz P. Gerken J. Schweer T. Yarza P. (2012). The SILVA ribosomal RNA gene database project: improved data processing and web-based tools. *Nucleic Acids Res.* 41 d590–d596. 10.1093/nar/gks1219 23193283 PMC3531112

[B71] Ramin M. Huhtanen P. (2013). Development of equations for predicting methane emissions from ruminants. *J. Dairy Sci.* 96 2476–2493. 10.3168/jds.2012-6095 23403199

[B72] Ramos A. F. O. Terry S. A. Holman D. B. Breves G. Pereira L. G. R. Silva A. G. M. (2018). Tucumã oil shifted ruminal fermentation, reducing methane production and altering the microbiome but decreased substrate digestibility within a RUSITEC fed a mixed hay – concentrate diet. *Front. Microbiol.* 9:1647 10.3389/fmicb.2018.01647 PMC6071481 30093888

[B73] Roehe R. Dewhurst R. J. Duthie C. A. Rooke J. A. Mckain N. Ross D. W. (2016). Bovine host genetic variation influences rumen microbial methane production with best selection criterion for low methane emitting and efficiently feed converting hosts based on metagenomic gene abundance. *PLoS Genetics* 12:e1005846. 10.1371/journal.pgen.1005846 26891056 PMC4758630

[B74] Saleem A. M. Ribeiro G. Yang W. Z. Ran T. Beauchemin K. A. Mcgeough E. J. (2018). Effect of engineered biocarbon on rumen fermentation, microbial protein synthesis, and methane production in an artificial rumen (RUSITEC) fed a high forage diet. *J. Anim. Sci.* 96 3121–3130. 10.1093/jas/sky204 29912357 PMC6095387

[B75] Scharen M. Drong C. Kiri K. Riede S. Gardener M. Meyer U. (2017). Differential effects of monensin and a blend of essential oils on rumen microbiota composition of transition dairy cows. *J. Dairy Sci.* 100 2765–2783. 10.3168/jds.2016-11994 28161182

[B76] Schären M. Frahm J. Kersten S. Meyer U. Hummel J. Breves G. (2018). Interrelations between the rumen microbiota and production, behavioral, rumen fermentation, metabolic, and immunological attributes of dairy cows. *J. Dairy Sci.* 101 4615–4637. 10.3168/jds.2017-13736 29454699

[B77] Seedorf H. Kittelmann S. Janssen P. H. (2015). Few highly abundant operational taxonomic units dominate within rumen methanogenic archaeal species in New Zealand sheep and cattle. *Appl. Environ. Microbiol.* 81:986. 10.1128/AEM.03018-14 25416771 PMC4292475

[B78] Singh K. Tripathi A. Pandya P. Parnerkar S. Kothari R. Joshi C. (2013). Molecular genetic diversity and quantitation of methanogen in ruminal fluid of buffalo (Bubalus bubalis) fed ration (wheat straw and concentrate mixture diet). *Genet. Res. Int.* 2013:980191. 10.1155/2013/980191 23862067 PMC3687512

[B79] St-Pierre B. Wright A. D. (2013). Diversity of gut methanogens in herbivorous animals. *Animal* 7(Suppl. 1), 49–56. 10.1017/S1751731112000912 22717175

[B80] Terry S. A. Ramos A. F. O. Holman D. B. Mcallister T. A. Breves G. Chaves A. V. (2018). Humic substances alter ammonia production and the microbial populations within a RUSITEC fed a mixed hay - concentrate diet. *Front. Microbiol.* 9:11. 10.3389/fmicb.2018.01410 30013529 PMC6036602

[B81] Thies J. Rillig M. (2009). “Characteristics of biochar: biological properties,” in *Biochar for Environmental Management: Science and Technology*, 1 Edn, eds Lehmann J. Joseph S. (London: Taylor and Francis Ltd.), 85–105.

[B82] Van Haarlem R. P. Desjardins R. L. Gao Z. Flesch T. K. Li X. (2008). Methane and ammonia emissions from a beef feedlot in western Canada for a twelve-day period in the fall. *Can. J. Anim. Sci.* 88 641–649.

[B83] Van Soest P. J. Robertson J. B. Lewis B. A. (1991). Methods for dietary fiber, neutral detergent fiber, and nonstarch polysaccharides in relation to animal nutrition. *J. Dairy Sci.* 74 3583–3597. 1660498 10.3168/jds.S0022-0302(91)78551-2

[B84] Yu L. Yuan Y. Tang J. Wang Y. Zhou S. (2015). Biochar as an electron shuttle for reductive dechlorination of pentachlorophenol by Geobacter sulfurreducens. *Sci. Rep.* 5:10. 10.1038/srep16221 26592958 PMC4655402

[B85] Zhou G.-W. Yang X.-R. Marshall C. W. Li H. Zheng B.-X. Yan Y. (2017). Biochar addition increases the rates of dissimilatory iron reduction and methanogenesis in ferrihydrite enrichments. *Front. Microbiol.* 8:589. 10.3389/fmicb.2017.00589 28428774 PMC5382251

[B86] Zhou M. Hernandez-Sanabria E. Guan L. L. (2009). Assessment of the microbial ecology of ruminal methanogens in cattle with different feed efficiencies. *Appl. Environ. Microbiol.* 75 6524–6533. 10.1128/AEM.02815-08 19717632 PMC2765141

[B87] Zhou M. Hernandez-Sanabria E. Guan L. L. (2010). Characterization of variation in rumen methanogenic communities under different dietary and host feed efficiency conditions, as determined by PCR-denaturing gradient gel electrophoresis analysis. *Appl. Environ. Microbiol.* 76 3776–3786. 10.1128/AEM.00010-10 20418436 PMC2893468

[B88] Zuo W. Chijioke O. E. Jinzhen J. Min W. Shaoxun T. Chuanshe Z. (2017). Investigation and manipulation of metabolically active methanogen community composition during rumen development in black goats. *Sci. Rep.* 7:1. 10.1038/s41598-017-00500-5 28341835 PMC5428682

