# Supplementary figures and images for: Effects of Hardwood Biochar on Methane Production, Fermentation Characteristics, and the Rumen Microbiota Using Rumen Simulation
Source: Front Microbiol. 2019 Jul 10;10:1534. doi: 10.3389/fmicb.2019.01534 (PMC6635593; doi:10.3389/fmicb.2019.01534)

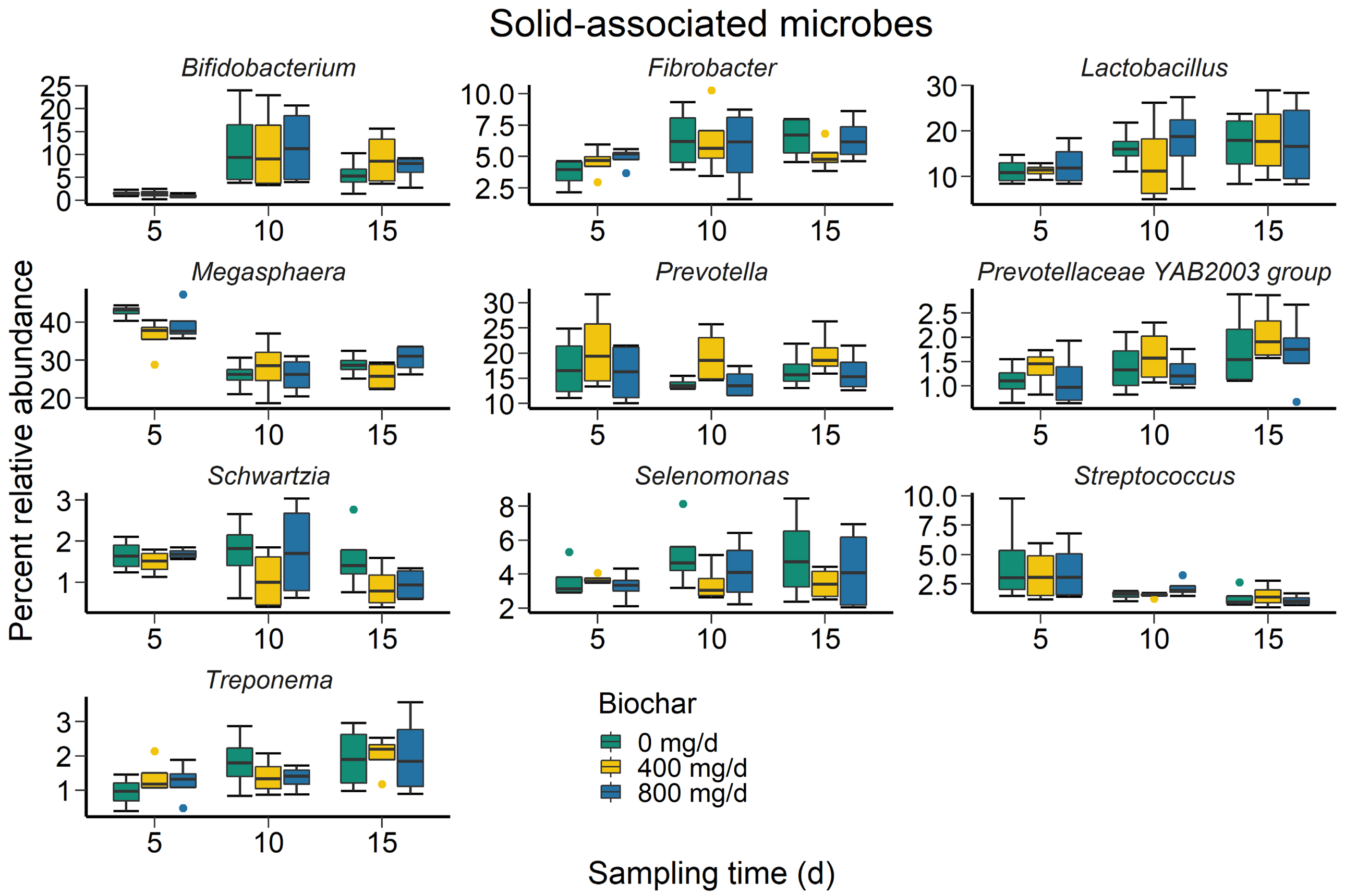

Supplement: FIGURE S1 — Box and whisker plots of the 10 most relative abundant bacterial genera in the solid-associated microbe (SAM) rumen samples by sampling time and biochar concentration. The box in the box plots indicates the interquartile range (IQR) (middle 50% of the data), the middle line represents the median value, and the whiskers represents 1.5 times the IQR. [file Image_1.TIFF]

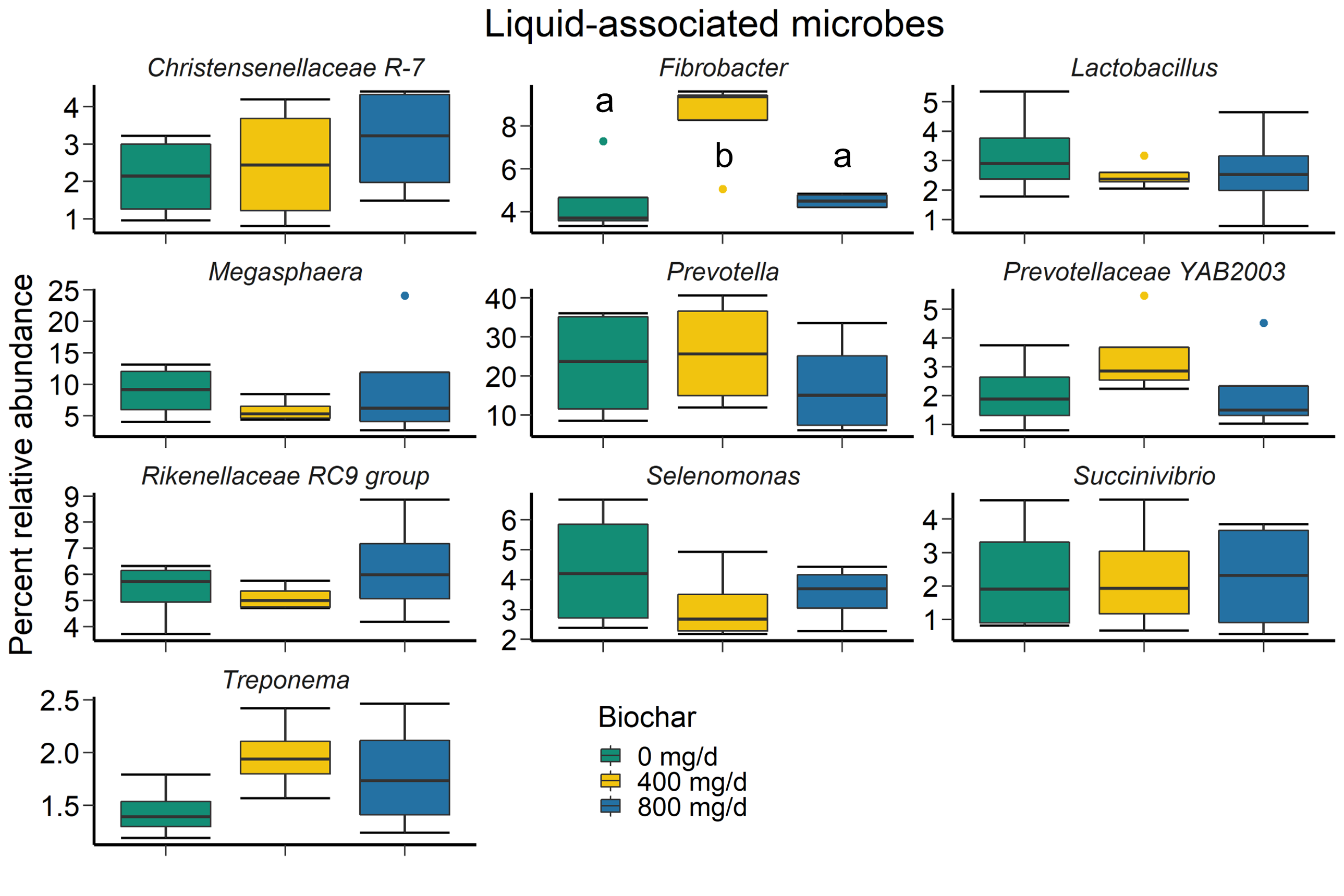

Supplement: FIGURE S2 — Box and whisker plots of the 10 most relative abundant bacterial genera in the liquid-associated microbe (LAM) rumen samples by sampling time and biochar concentration. The box in the box plots indicates the interquartile range (IQR) (middle 50% of the data), the middle line represents the median value, and the whiskers represents 1.5 times the IQR. Different lowercase letters indicate significantly different means (P ≤ 0.05). [file Image_2.TIFF]

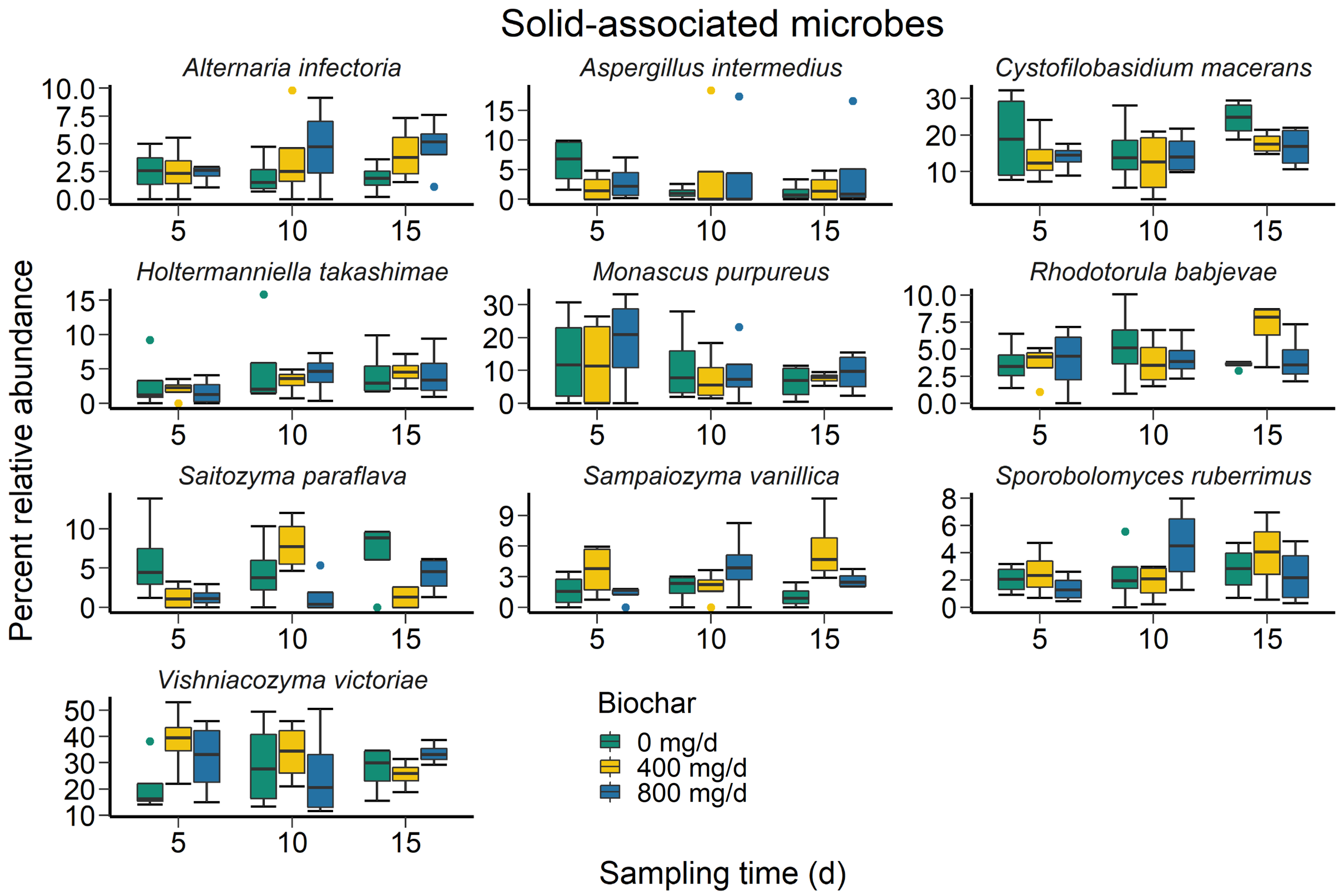

Supplement: FIGURE S3 — Box and whisker plots of the 10 most relatively abundant fungal species in the solid-associated microbe (SAM) rumen samples by sampling time and biochar concentration. The box in the box plots indicates the interquartile range (IQR) (middle 50% of the data), the middle line represents the median value, and the whiskers represents 1.5 times the IQR. Different lowercase letters within each sampling time represent significantly different means (P < 0.05). [file Image_3.TIFF]

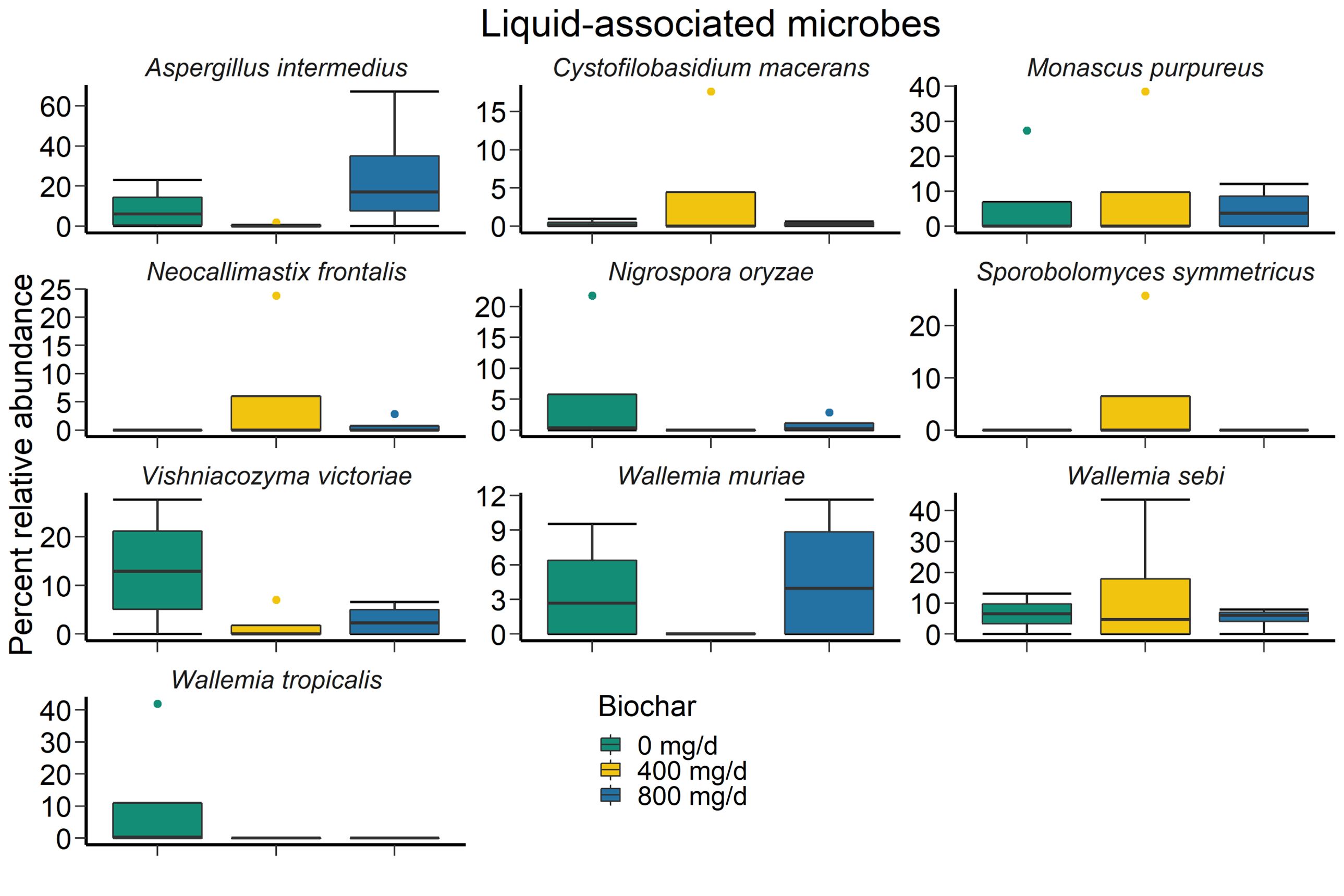

Supplement: FIGURE S4 — Box and whisker plots of the 10 most relative abundant fungal species in the liquid-associated microbe (LAM) rumen samples by sampling time and biochar concentration. The box in the box plots indicates the interquartile range (IQR) (middle 50% of the data), the middle line represents the median value, and the whiskers represents 1.5 times the IQR. [file Image_4.TIFF]
